# Co-designing accessible and inclusive patient information resources for gastrointestinal endoscopy using Patient and Public Involvement (PPI) and Universal Design for Learning (UDL) principles

**DOI:** 10.1371/journal.pone.0333874

**Published:** 2025-10-16

**Authors:** Seán Fennessy, Liam Mulcahy, Brian O’Donnell, Hugh Mulcahy, Muirne Spooner, Edel McDermott

**Affiliations:** 1 School of Medicine, University College Dublin, Dublin, Ireland; 2 Department of Gastroenterology, St Vincent’s University Hospital, Dublin, Ireland; 3 Department of Medicine, Royal College of Surgeons in Ireland, Dublin, Ireland; JSPS Government Homoeopathic Medical College, INDIA

## Abstract

Co-designing accessible and inclusive patient information resources for gastrointestinal endoscopy using Patient and Public Involvement (PPI) and Universal Design for Learning (UDL) Principles.

**Introduction:**

Patients undergoing GI endoscopy can experience anxiety before their procedure, for numerous different reasons, including ineffective patient education resources received beforehand. Paper-based information leaflets are insufficient to accommodate for the diverse way in which people access, consume and process information. Public and patient involvement (PPI) and Universal Design for Learning (UDL) are two well-described pedagogical principles that strive to optimise patient-centred care and inclusivity.

**Objectives:**

Our aim was to apply these principles to design more effective and accessible patient education materials, improving the health literacy of our patients. Working with patient partners, we identified the need to develop high-quality and trustable video resources for patients, that would be available on our hospital website. These videos were co-designed by patients and other key stakeholders.

**Results:**

We used techniques such as storyboard development, the UDL educational principles of representation, engagement and expression, as well as the individual expertise of our stakeholder panel members to achieve appropriate and accessible information for our patient cohort. The development phase was an iterative process, with feedback and input from patient partners and other stakeholders playing a crucial role in prompting necessary adjustments for accuracy and patient understanding. Our project is the first guide in combining both PPI and UDL principles in the development of patient information and education materials.

**Conclusion:**

By involving patients and other key stakeholders as partners, we improved the relevance and quality of our patient information content. Identification of patient partners and appropriate other stakeholders is an important initial step when co-designing patient information resources. The use of UDL in the co-design process allows for a structured approach to creating accessible content, highlighting important steps that otherwise may be overlooked by team members. Formal assessment of the impact of these co-designed videos, through quantitative and qualitative methods, will be assessed as part of a larger study.

## Introduction

In today’s online world, the availability of medical information can be overwhelming for patients and clinicians alike. The rise of digital misinformation can significantly increase poor health choices and outcomes [1,2]. Limited health literacy and inaccessible communication materials are barriers to patient engagement and education [3–5]. Limited health literacy affects a large proportion of adults, with 47% of Europeans having “insufficient or problematic” health literacy in a 2012 EU Health Literacy Survey [6]. The WHO mandate for health literacy has identified that media serve as a “critical platform for health literacy messaging… meeting an ethical threshold for accuracy to support, rather than subvert, people’s right to health” [7]. This is a call to action for healthcare professionals in recognising that the health information they create must be centrally focussed on supporting patients to make informed choices, accommodating various levels of health literacy. This mandate has been linked to the UN Agenda for Sustainable Development [8].

Digital initiatives can lessen healthcare inequality by improving accessibility, but it is critical to ensure that patients can comprehend the information that is made available [9,10]. Many online patient information sources are reported as low quality [11]. A 2022 study of 3,000 papers showed that just over 1-in-5 papers had any patient involvement in their design process [12]. Patient co-design is essential for creating healthcare services that truly meet patient needs. By leveraging the patient’s voice and unique perspectives and experiences, we can tailor services appropriately [13].

In parallel to acknowledging the essential role of patients in co-creating resources to support their care, there is increasing acknowledgment of diversity of learners in general, i.e., that people learn through different means. There are a number of plain language and accessibility guidelines, they often exist in isolation rather than being consolidated into a unified framework [14,15]. This can leave developers of resources with fragmented direction. Universal Design for Learning (UDL) is a set of principles for curriculum development, that is becoming increasingly popular in educational contexts. UDL is centred around improving the learning experience for all by acknowledging and addressing the diversity amongst the population.

While this seems ideally placed for patients, there is little evidence of their application in patient education resources. These concepts are linked in our work to recognise that patients are also learners, and applying the educational principles of UDL in combination with patient co-creation will provide resources that encompass the patient voice and will also be accessible to wide-ranging patients.

PPI is when research is carried out in conjunction with members of the public, rather than done solely from a healthcare professionals’ perspective [16]. PPI in research has been registered as best practice and is now a core component for granting of research funding in some countries [17]. PPI can improve study enrolment and promote dissemination of study results [18,19]. There are seven key PPI principles, which are outlined in Table 1. A reporting framework, Guidance for Reporting Involvement of Patients and the Public (GRIPP2), was developed to standardise reporting in PPI studies, improving quality, transparency and consistency [20].

**Table 1:** PPI Principles.

Principle	Definition	In practice
Empowerment and power sharing	Involving patients and the public as soon as possible in funding decisions, design and production decisions related to research.	SF and EMcD brainstormed the appropriate stakeholders. We identified the following groups this way: Patients, an educationalist, plain English guidance expert, a member of the national quality and patient safety office and a patient representative group. After consulting with these groups, we broadened stakeholders to also include endoscopy administrative staff, the hospital communications manager, and the clinical audit chair.
Collaboration and partnership	Including PPI partners throughout the research lifecycle. Expectations should be clarified from the beginning. PPI aims to create a true partnership, where all contributions are valued and respected equally.	All stakeholders were provided with the research proposal and invited to contribute to its refinement. All stakeholders met regularly online with an agenda provided in advance and minutes/actions afterwards by SF.
Equity and inclusion	Identify and remove potential barriers to inclusion and creating entry points throughout the lifecycle of research. PPI aims to have real co-design and partnership, that values people’s different opinions, experiences, abilities, backgrounds and expertise equally.	Meeting times and dates were decided using online polling software, to choose the time that suited most stakeholders. Meetings were held in person and via Zoom to ensure maximum opportunity to attend for all stakeholders. Free and open discussion was facilitated by the panel chair, EMcD.
Flexibility	Recognise the time and other commitments involved in PPI and that is enacted in the research programme.	Meeting times and dates were decided using online polling software, to choose the time that suited most stakeholders. Meetings were held in person and via Zoom to ensure maximum opportunity to attend for all stakeholders.
Transparency	PPI calls for clear and open communication between researchers and PPI partners about research decisions and progress.	All stakeholders were given a clear, written outline of the proposed project before agreeing to join the panel. An agenda was sent before each meeting, as well as any items to review. Meeting minutes were sent within 24 hours of a meeting, for review by the panel.
Respect	Recognition of the roles, knowledge, insights, experiences, strengths, limitations and contributions across the research team and PPI partners. The diversity of an expanded team will benefit the research.	Each person’s expertise was listened to by all panel members, and everyone’s contribution was acknowledged. Input was encouraged from all stakeholders. Any differences of opinion were discussed by the panel and a consensus decision was reached. Our adherence to the other six principles also demonstrated our respect for all stakeholders.
Trust	Reciprocation of trust is important in a partnership and should be consistently worked on. Each member’s opinion should be listened to and confidentiality should be respected.	When stakeholders shared sensitive or personal information, this was handled with discretion and respect by all stakeholders. Frank discussion by all stakeholders about their own experiences created a safe and trusting environment for other members.

PPI and its principles can be implemented in several different process throughout the research life cycle. The most common process used in PPI, is co-design. This is where patients, other key stakeholders and health professionals collaborate to design trials, specific interventions or resources. Patients are usually actively involved in giving design input and feedback, but the overall process is steered by healthcare professionals. The expertise and ideas from patients are essential in the design process, but ultimately, the decision to include/exclude ideas lie with the healthcare professionals.

Co-production is seen as a longer-term partnership between patients and healthcare professionals. Patient involvement is throughout the project, from conception of ideas and design to evaluation of the project at its completion [21]. Co-production includes service users in the implementation of solutions that have already been agreed upon, for described problems [22].

### Objectives

The aim of our paper is to outline how to include patient’s unique perspectives and opinions, to co-design and co-produce patient information resources (PIR), which include inclusive learning designs, taken from UDL principles. We utilised these principles throughout our design for PIR videos for our outpatient endoscopy patients, to be used as an addition to our paper-based leaflets. We will detail how we included these principles in a comprehensive and pragmatic manner for our project, as well as through the GRIPP2 report.

## Methods

### Harnessing public and patient partnership in content co-design

**1) Establishing a stakeholder panel**: Significant planning is required to potentiate partnership in creating the high-quality educational resources. When working with patients and members of the public in co-designing patient information resources, we began with assembling a panel of key stakeholders, whose expertise and insights are critical to progression of the project. For example, in this project, doctors, endoscopy nurses, endoscopy administration, patients, educational specialists and a plain language expert were included. This balanced mix of knowledge and experience allowed for the capture a diverse range of patient experiences and professional insights. This purposeful sampling method was essential for selecting stakeholder members, ensuring the inclusion of individuals with relevant expertise, experiences, and perspectives. This diversity helps identify practical challenges, service gaps, and opportunities for enhancing patient care and operational efficiency. Purposeful sampling supports the development of recommendations that are both evidence-informed and grounded in real-world experience, increasing the relevance, acceptability, and impact of proposed improvements.

The PPI principles of empowerment and power-sharing and collaboration and partnership were used to assemble our key stakeholder panel. Our first stakeholder panel meeting was held on the 24^th^ January 2023, with the final feedback from our stakeholders collected on the 6^th^ March 2024.

**2) Aligning goals:** When recruiting potential panel members, it’s essential to provide them with an overview of the project, including the rationale behind its aims and objectives, as well as how you intend to achieve them. This ensures their perspectives are aligned with the project’s goals. However, it’s not necessary for them to share the same vision as the researchers, as the diversity of opinions is key to successful co-design. For example, in our initial proposal we planned to disseminate our PIR via email. After sharing with our collaborators, they indicated whilst they would agree, they felt that the majority would prefer to be contact via text message or included in the pre-procedure information. A major concern amongst our stakeholders is that patients may be worried regarding ‘spam’ emails and would be less likely to click on an email link. This interaction with our collaborators incorporates the PPI principles of trust, equity and inclusion, transparency and flexibility.
**3) Setting the content and format using storyboarding**: Storyboarding is a technique, which allow key concepts to be visualised and plan the sequence of information in the video [23]. Storyboard development can also be useful when designing non-video projects, such as leaflets, booklets, etc. This facilitates seamless collaboration as it provides a common vision for group members as a starting point. This allows cohesive work when giving feedback and for an efficient recording and editing process for each of the videos. In our project, we discussed the theme and content for each video as a stakeholder group. Then the author, SF, used this input to design storyboards for each video, which were circulated to the stakeholders via email for review. Each storyboard was reviewed again as part of our stakeholder meeting, where edits were made accordingly. For those unable to attend the meeting, they were asked to give their feedback via email beforehand, so that they could be included in the editing process. This co-design process included all seven PPI principles.
**4) Testing and feedback**: Before finalising the PIR, a comprehensive testing phase was initiated with our patient representatives and stakeholder panel, in keeping with the principle of collaboration and partnership. Our initial video was shown to the stakeholder committee in a group viewing and comments and first impressions were noted. Each member was given a link to watch it individually so that they could provide further feedback in their own time and given the opportunity to provide feedback in a group and individual setting. Individual feedback was through a home-designed feedback form, with input from the stakeholder panel.

### Ethical approval

Ethical approval was obtained via the St Vincent’s Research Ethics Committee – approval number RS22–051. Ethics approval letter has been uploaded. Ethics approval covers the entire project, including the video development aspect. The video development process did not involve gathering or recording personal, identifiable, or sensitive information about stakeholders. Contributions were focused on general insights, feedback or expertise, rather than individual experiences or characteristics that could lead to identification. No patients or stakeholders were recorded as part of the recording series. Only staff members were recorded, and they signed hospital consent forms for recording audio/video/photography. No formal consent was required for membership of the stakeholder panel and ground rules for the process were established at the first stakeholder group meeting.

### Universal design for learning (UDL) principles

The three primary principles of UDL are described in Table 2:

**Table 2:** UDL Principles.

Principle	Description	In practice
Multiple means of engagement	The “why” of learning. Provide options for: Recruiting interest – e.g., optimising individual choice and autonomy; optimising relevance. Sustaining effort and persistence – e.g., fostering collaboration and community, highlighting importance of goals and objectives. Self-regulation – e.g., promoting expectations and beliefs that optimise motivation.	The UDL principles state that we should “allow learners to participate in the design of classroom activities and academic tasks... in setting their own personal academic and behavioural goals” [24]. We have elevated this requirement by utilising co-design principles to ensure maximum input. Many videos focusing on pre-endoscopy information are more than 5 minutes in duration and this can decrease engagement, as well as information retention. We have divided our content into smaller videos, less than 3 minutes in duration [average length 113 seconds] so that patients are more likely to watch the videos as they will only watch the videos that they feel are relevant to them. This will improve their chances of retaining the information as we know from previous studies that increasing video length reduces retention [25].
Multiple means of representation	The “what” of learning. Provide options for: Perception – e.g., offering methods to customise the visual display of information and auditory information. Language and symbols – e.g., clarifying vocabulary and using multiple media forms to illustrate information. Comprehension – e.g., highlight patterns, critical features and big ideas. Maximise information transfer and generalisation.	By offering digital content, along with the print material, we were able to significantly improve our educational offering. The importance of plain-language experts ensured that the content information was accessible to the wider public. Hosting the information on our hospital website, gives patients confidence in the quality of our information and security of the website. We primarily used a voice-over to narrate our videos, which helps to control the volume, sound quality and rate of speech. We embedded subtitles into each video and a PDF with the text of the videos is also available under each video. These subtitles/PDF can be translated into other languages through online resources for the patient’s reference. We used Calibri font to make letters appear less crowded, to help patients with dyslexia. During our co-design process, we were advised to use black text on a white background for important information, to emphasise the importance. We avoided using capital letters to stress importance, as this can imply that we are shouting. Another example is how we used large red ‘X’ was placed through images of foodstuffs that patients were not supposed to eat prior to a colonoscopy (see Fig 1).
Multiple means of action and expression	The “how” of learning. Provide options for: Physical action – e.g., varying methods for response and navigation. Optimising access to tools and assistive technologies. Expression and communication – Use multiple media for communication. Executive functions – Facilitate managing information and resources and enhance capacity for monitoring progress.	Video platforms allow for individuals to adapt the speed, sound, quality, and size of the video to their own preferences. The hospital website, which hosts the videos and other information resources is compatible with screen readers, increasing accessibility for those with visual impairment. The PDFs accompanying each video can be digested using text to speech technologies. By using plain language in our videos and subsequently our PDFs, it reduces the complexity of the text and increases the accuracy of online translation tools. There are multiple accessibility testing tools available online or within software programs, such as Microsoft’s Accessibility Checker, that can assess the content within your information resources and ensure it is easier for people with disabilities to read.

**Fig 1:**
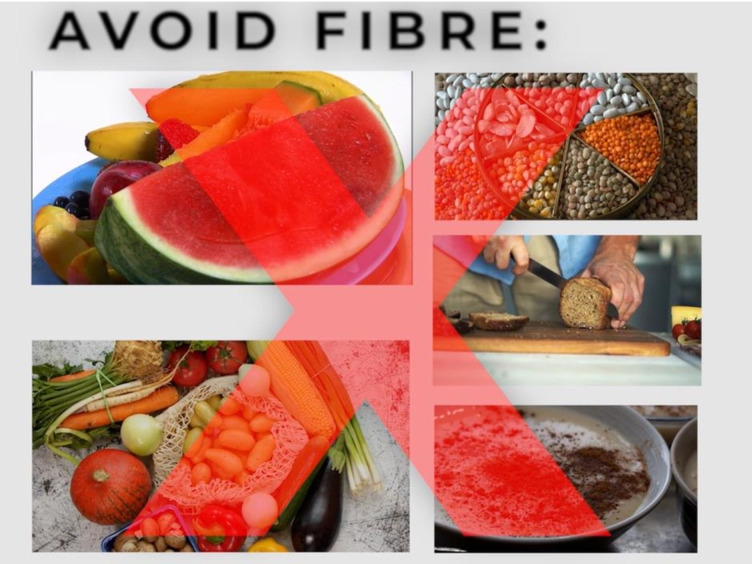
Patient video screenshot. A screenshot from one of the patient education videos with a large red ‘X’ to emphasise which foods to avoid.

**a) Multiple means of representation** – patients will differ in how they perceive and comprehend information and so offering content through different modalities is important to reduce barriers to patient education [24]
**b) Multiple means of action and expression** – patients will differ in how they interact with learning materials and so having a mechanism to navigate and control accessibility ensures equal opportunities for patients [24].
**c) Multiple means of engagement** – not only will different patients have different preferences for what sources engage them from a learning perspective, but individual patient preferences can also change over time, or for different learning circumstances.

The ethos of UDL is to promote an inclusive educational environment and is aimed at optimising teaching and learning for all individuals by addressing the diverse learning requirements amongst the general population. This has parallels with inclusion health which refers to groups of people who have multiple overlapping risks for poor health [26]. In creating resources that implement UDL, this work aimed to provide accessible high-quality information to diverse patient populations, as we recognise that patients are not one homogenous cohort.

## Results

**Fig 2:**
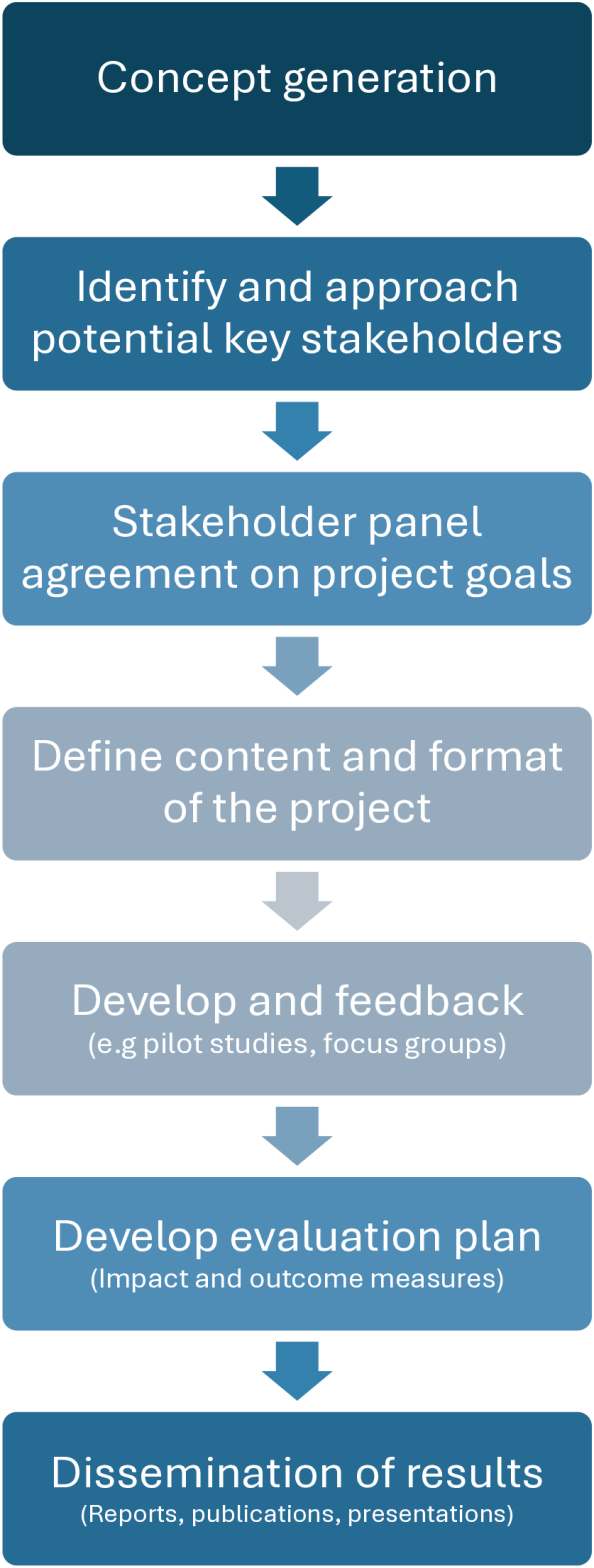
Co-design project flowchart. A visualisation of the different steps and timeline in our co-design project.

The co-design process unfolded across several distinct stages, each contributing to the development and refinement of the final outcome. A visual summary of the overall project structure and progression is presented in Fig 2.

### The stakeholder panel

Ensuring the appropriate range of expertise amongst the panel was a very important consideration for us. Bring together people from relevant clinical, technical, and educational backgrounds. Our first step was to form a stakeholder panel comprising key individuals in outpatient endoscopy (S1 Table). This included healthcare professionals, patients, educators, a plain-language expert, administrative staff, and a professional videographer. The panel included 14 members (4 female, 3 non-White Irish) and included a diverse mix of clinical and administrative experience – the endoscopy department nurse managers and administrative lead, two consultant gastroenterologists, a non-consultant hospital doctor, two medical students, the hospital’s communications manager and head of clinical audit, as well as the heads of IT and data protection. The two patients included one male patient in his thirties, and a female patient in her seventies. This approach helped to ensure that the educational materials are relevant, understandable and culturally sensitive.

Involving patients or members of the public is not the only criterion to satisfy PPI engagement, the opinion of ancillary staff members is also an important consideration. For example, our project was fortunate to have excellent insights from our check-in and administrative staff. This allowed us to clarify certain points within our PIRs, such as the difference between “check-in time” and time of endoscopy and that different procedures run concurrently, which is why certain patients may be called sooner.

### Aligning goals

After assembling a stakeholder panel, the authors devised a broad outline of the project and communicated this to the panel members individually, in advance of the first stakeholder meeting, with agreement of aims and deliverables at the first panel meeting.

### Setting the content and format using storyboarding

The authors wanted to offer digital PIRs, in keeping with our department’s commitment to sustainability and reducing our paper/postage burden. Our patient partners agreed with this option, but also recommended keeping the current paper based PIRs, as internet access or digital literacy is not universal.

Together we decided to offer short, easy-to-understand videos, which would be the cornerstone of our digital offering. Each video would focus on a different topic within the endoscopy experience, rather than having longer videos containing multiple topics, which may not be of interest to some patients. The panel believed that producing shorter videos focusing on one topic each, would empower patients to select the specific content they wished to explore, thereby optimising their level of engagement. For thevideos, the panel decided that the videos should be informative, but not serious. The group wanted to convey a sense of friendliness and calmness, without viewers thinking that we were making light of the procedures. The patient representatives, as well as thecorporate communications manager had significant input into this aspect.

A comprehensive list of topics was compiled during a monthly stakeholder meeting and can be seen in Table 3. Existing patient information leaflets were scrutinised to ensure no critical information was overlooked and to ensure consistency of available information from our department. Essential information from each PIL was tabulated to ensure no key points were missed. In certain cases, information leaflets were updated in keeping with the latest guidelines and medication changes. The authors meticulously reviewed and tabulated essential information, forming the foundation for the subsequent video content.

**Table 3:** Video Contents List.

OGD overview	2:03
Colonoscopy overview	2:09
Conscious sedation	1:58
Endoscopy department and how to get there	0:58
“A typical day”	1:08
Bowel preparation – how to take it and tips (morning and afternoon videos)	2:27
Peri-colonoscopy diet	2:26
Follow-up	1:05

**Fig 3:**
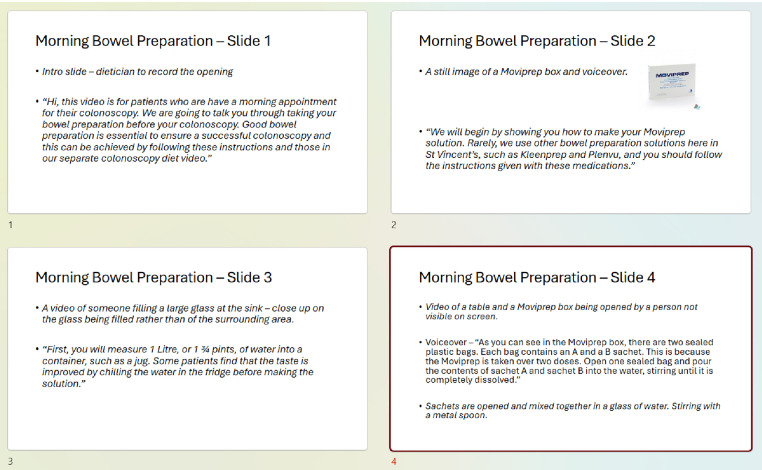
Storyboard example. An example of some of the storyboarding slides used as part of the study.

For each of the videos, a separate storyboard was created using Microsoft PowerPoint (see Fig 3). We included the script, as well as directions for the filming and editing process. These directions include instructions regarding on-screen text and insertion of animations/images. There are specific storyboarding software packages and templates available also.

A video script was devised by the authors for each video, with input from our stakeholder panel. Each script was reviewed by our plain language expert. The video script was written after we developed a storyboard for the video, to ensure all important information was included and cross-referenced with our existing literature to ensure consistency on information.

The plain language and accessible principles recommend offering multiple choices to learners to encourage the development of “self-determination, pride in accomplishment and increase the degree to which they feel connected to their learning” [24]. To augment the video offerings, we have included ‘frequently asked question’ topics to our website and links to high-quality, trustworthy, external resources (both video and text-based). Calibri font was used to make letters appear less crowded, to help patients with dyslexia. Table 2 shows some examples of how we specifically used UDL principles in our work.

### Testing and feedback

Before finalising the videos, a comprehensive testing phase was initiated. An initial video was shown to the stakeholder committee in a group viewing and comments and first impressions were noted. Each member was given a link to watch it individually so that they could provide further feedback in their own time and given the opportunity to provide feedback in a group and individual setting.

One example was that the pen we used to compare the endoscope with was too wide and so was a false representation of the endoscope width, potentially leading patients to think the camera was much smaller than reality, which may cause them distress on the day. This scene was then re-filmed using a more appropriate size pen. Table 4 contains a selection of comments by panel members.

**Table 4:** Selection of feedback from panel members.

*Patient representative 1; Colonoscopy video*: “it is important to show the underwear before the procedure, as that can be a shock to patients… comparing the width of the tube to a larger pen is deceitful to the patients” *Patient representative 2; Bowel preparation video: “*I thought it was good, clear and the information was given at a good pace. I thought the video of the person preparing the solution with the presenter type person was a good touch and makes it a bit more personable” *Administrator, Endoscopy Unit; Discharge instructions video:* “we should explain our virtual follow up clinic process more… putting definitive timeframes on results can lead patients to becoming worried and stressed if they do not arrive on time” *Hospital communications manager; Peri-colonoscopy diet video:* “a good flow, very informative… need to change the some of the images and include a large red ‘X’ through the food so that patients do not get confused”

The panel’s feedback was utilised to co-design the final videos. Panel members were sent department-designed feedback forms about the videos (S2 Table). These were then reviewed by the lead authors and discussed with the video production team.

Feedback was analysed by SF, EM and MS. Each independently read and re-read the data to familiarise themselves with the documents. Codes were then identified which were grouped into themes. These were discussed and revised until consensus was reached. Three main themes were identified: Inclusivity, Accessibility and Patient-centredness. Almost all stakeholders prioritised a deliberate effort to involve and represent the perspectives, needs, and experiences of a wide range of individuals:

*“I think it’s also helpful in giving patients a realistic idea of waiting times versus thinking they’ll be in an out in in an hour or two.”*

They also commonly discussed removing barriers that might prevent individuals from understanding or engaging and provided examples on earlier versions where accessibility could be improved, e.g., moving subtitles, slowing pace, replacing words and phrased with more easily recognised ones, etc. Feedback from the literacy expert included:

“You will note posters…”

Note is not a common word for everyone.

Better to use: “You will see posters ….”

And removing the word “unforeseen” when referring to potential delays within the department, to make our phrasing easier to understand.

The third theme was patient-centredness. Stakeholders provided feedback that focused on what is likely to be meaningful to patients rather than clinicians, e.g., one identified that patients need to know if they need someone to stay overnight with them after having conscious sedation versus if they choose an “awake” procedure. Or even navigating their way around the hospital in general, with one of the patients commenting:

*“Showing the waiting area is helpful, the hospital itself can be a bit of a maze! Even at around 50 seconds in the voice over calls the area the pre-endoscopy bay and that’s helpful to know.”*

These three themes tie in with the overall goal of improving health literacy.

The videos are publicly available on the hospital website, https://www.stvincents.i.e.,/departments/endoscopy/patient-information-resources/.

An important final aspect of the video design development was ensuring that follow up studies were in place to evaluate the videos so that we can improve upon them. We have already assessed the patient experience prior to the implementation of these videos. We plan to assess the patient experience, through qualitative and quantitative forms, after the introduction of these videos and take the feedback on-board, especially related to accessibility of the materials. This is an extra layer to our co-design process, by incorporating post-hoc analysis and feedback for editing our current videos, as well as providing a foundation for future video design.

We will also track how many patients access and view the video to understand which sections are most popular and where viewers may drop off.

## Discussion

Developing patient information resources requires a comprehensive approach involving stakeholders and considering diverse patient needs. Incorporating two well-established theoretical frameworks (PPI and UDL) is a novel approach in applying best evidence from the wider educational landscape to patient resources, with the aim of optimising healthcare delivery.

As this paper has demonstrated, both UDL and PPI principles ensure that inclusivity and flexibility of access and understanding are core features of both pedagogical principles, meaning they can be used in virtually all settings and scenarios worldwide. For example, in countries with diverse cultural and linguistic populations, following UDL principles ensures equitable access to health information and services. Following PPI principles allows for the design of services or literature that reflect service user priorities and specific cultural beliefs. The use of these principles is not confined to high-income countries. For example, a systematic review has shown that community engagement and empowerment among female sex workers in low and middle-income countries can effectively prevent the spread of HIV infection [27].

However, PPI is underdeveloped in low and middle-income countries and so appropriate frameworks and guidance is needed in these instances [28]. This study is especially relevant to these countries as it highlights the importance of following PPI principles, but also the UDL principles to reach multiple populations within a country, through its patient-centred approach. Whilst not targeting patients, but healthcare professionals, the United Nations SURGhub is an example of digital education content that is successfully delivered by providing freely accessible and culturally relevant content in among low and middle income countries [29].

## Limitations

Whilst this research has been meticulously devised and with the aim of improving understanding of the UDL and PPI frameworks, there are some potential limitations. Training and implementation of these pedagogical principles can require funding and dedicated time and human resources. Certain healthcare situations may lack the infrastructure or staff training needed to support meaningful change. However, this is why we have devised this manuscript, so that people can understand that this can be effectively achieved without the use of significant resources.

There is currently no formal, validated usability metric specifically designed to measure the impact of Patient and Public Involvement (PPI) and/or Universal Design for Learning (UDL) in research contexts. As a result, researchers often rely on qualitative approaches to assess how these principles influence research design, inclusivity, and outcomes. While these methods can offer rich, contextual insights into participants’ experiences and the perceived value of PPI or UDL, they are also limited by issues such as subjectivity, potential bias, and challenges in generalising findings. The lack of standardised metrics makes it difficult to compare results across studies or systematically assess improvements over time. This highlights a significant gap in the field and underscores the need for the development of robust, validated tools that can capture the usability and impact of PPI and UDL in research in a more consistent and measurable way. However, the questionnaire planned in the evaluation phase will have usability questions taken from a patient-reported experience measure (PREM).

Finally, the use of purposeful sampling in the recruitment of stakeholders, gave a representative sample of stakeholders, but there is the possibility of selection bias. Just under 30% of our stakeholder group was female, which is lower than the approximately 50% female representation in our patient population. Unfortunately, the hospital does not routinely collect ethnicity, nationality or language data limiting the ability to assess how well reflects the broader patient demographics. An observational study done in the department in 2023, showed that 84.7% of respondents to a questionnaire were white Irish, which would be in keeping with the stakeholder panel representation.

### Evaluation of impact

A GRIPP2 report was completed and can be seen in S3 Table, in keeping with international standards for all PPI reporting [30].

The initial study to test the feasibility of the questionnaire has been conducted, with similar scores in both groups (n = 9 in both groups) thus far in our small sample size [Welch’s t-test: pre-endoscopy p value 0.17; during/post-endoscopy p value 0.33; overall p value 0.11]. Focusing on comprehension-specific questions, there is no difference between those who received the patient information videos as well as the usual standard of care, versus those who received paper-based standard of care information leaflets alone (p = 0.474). This could be due to the small sample size, the videos themselves or difficulties measuring smaller improvements due to the ceiling effects associated with questionnaires. This will be explored and explained further when the study is completed.

The authors intend to employ the Newcastle ENDOPREM™, a validated and endoscopy-specific PREM, to quantitatively assess differences in patient experience between those who receive these videos, as well as the standard of care, to those who receive standard paper-based information only, prior to undergoing endoscopy [31]. This questionnaire was chosen as it is validated in an outpatient endoscopy setting and include comprehension specific questions.

By comparing patient-reported experience scores across these groups, the study aims to determine whether video-based information delivery leads to measurable improvements in the overall endoscopy experience. To complement this quantitative assessment, qualitative data will be collected through semi-structured interviews and focus groups. This mixed-methods design will provide a comprehensive evaluation of the intervention’s impact, capturing both measurable outcomes and in-depth insights into patient perceptions and preferences. The full mixed-methods study will be published in due course.

## Conclusion

This journal article outlines a strategic initiative within a larger project aimed at improving outpatient endoscopy patient education through digital media. The meticulous steps taken, from stakeholder engagement to video production, reflect a commitment to inclusivity and patient-centred care. Ongoing evaluation and feedback mechanisms ensure continuous improvement, contributing to the overall success and sustainability of the digital service.

## Supporting information

S1 Table List of stakeholder panel members. A list of all the stakeholder panel members and their roles.
(DOCX)

S2 Table Stakeholder feedback form. A copy of the sample stakeholder feedback form.
(DOCX)

S3 Table GRIPP 2 report. The completed GRIPP 2 report for this study.
(DOCX)
