# Supplementary material for: Co-designing accessible and inclusive patient information resources for gastrointestinal endoscopy using Patient and Public Involvement (PPI) and Universal Design for Learning (UDL) principles
Source: PLoS One. 2025 Oct 16;20(10):e0333874. doi: 10.1371/journal.pone.0333874 (PMC12530560; doi:10.1371/journal.pone.0333874)
Supplement: S1 Table — A list of all the stakeholder panel members and their roles. (DOCX) [file pone.0333874.s001.docx]

## **Supporting Information**

### **S1 Table. List of stakeholder panel members.**

| ***Consultant Gastroenterologist*** | Project lead, consultant gastroenterologist in SVUH. Main areas of interest are general gastroenterology, having led on digital innovation in gastroenterology, and IBD, which was the focus of their PhD. |
| --- | --- |
| ***Research Registrar*** | Research registrar, MD candidate. |
| ***Nurse Manager*** | Clinical nurse manager in the endoscopy unit, SVUH. |
| ***Medical Student 1*** | Video production expertise |
| ***Medical Student 2*** | Video production expertise |
| ***Nurse Unit Manager*** | Endoscopy unit nurse manager |
| ***Consultant Gastroenterologist*** | Clinical professor, UCD; consultant gastroenterologist. Specialist in endoscopy and colorectal cancer. |
| **Clinical Audit** | St Vincent’s Healthcare Group Head of Clinical Audit. |
| ***Head of communications*** | St Vincent’s Healthcare Group Head of Corporate Communications. |
| ***Patient representative 1*** | Female; 70s; legal professional. |
| ***Patient representative 2*** | Male; 30s; small business owner. |
| ***Administration Manager*** | Administration, endoscopy unit. |
| ***IT Lead*** | IT. |
| ***Data Protection Officer*** | Data protection officer. |
