# Supplementary figures and images for: Co-designing accessible and inclusive patient information resources for gastrointestinal endoscopy using Patient and Public Involvement (PPI) and Universal Design for Learning (UDL) principles
Source: PLoS One. 2025 Oct 16;20(10):e0333874. doi: 10.1371/journal.pone.0333874 (PMC12530560; doi:10.1371/journal.pone.0333874)

## **Supporting information**

### **S2 Table. Stakeholder feedback form.**


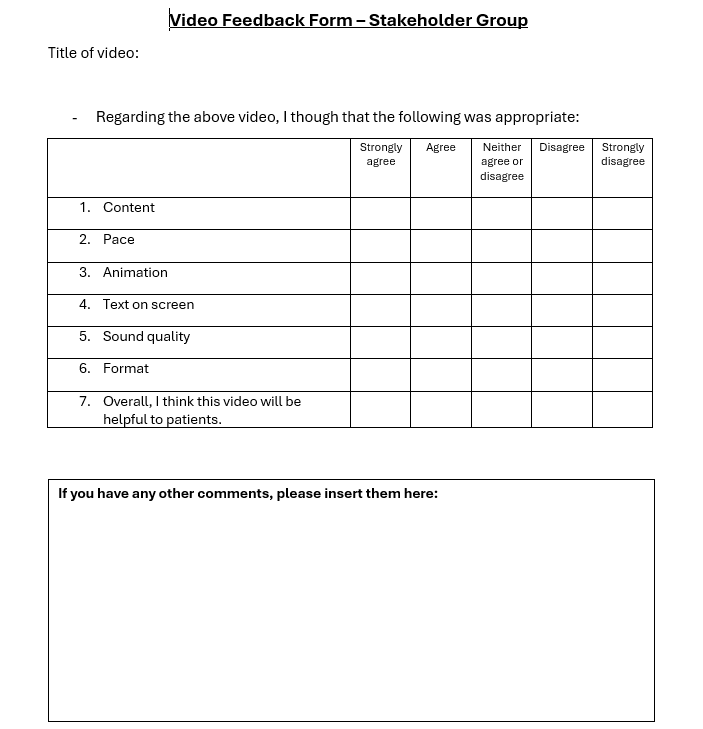

Supplement: S2 Table — A copy of the sample stakeholder feedback form. (DOCX) [file pone.0333874.s002.docx]
