# Supplementary material for: Co-designing accessible and inclusive patient information resources for gastrointestinal endoscopy using Patient and Public Involvement (PPI) and Universal Design for Learning (UDL) principles
Source: PLoS One. 2025 Oct 16;20(10):e0333874. doi: 10.1371/journal.pone.0333874 (PMC12530560; doi:10.1371/journal.pone.0333874)
Supplement: S3 Table — The completed GRIPP 2 report for this study. (DOCX) [file pone.0333874.s003.docx]

## **Supporting information**

### **S3 Table. GRIPP 2 report.**

| Section and topic | Item | | Reported on page No |
| --- | --- | --- | --- |
| Section 1: Abstract of paper | |  | |
| 1a: Aim | Report the aim of the study | | 3 |
| 1b: Methods | Describe the methods used by which patients and the public were involved | | 3, Table 1 |
| 1c: Results | Report the impacts and outcomes of PPI in the study | | 3 |
| 1d:Conclusions | Summarise the main conclusions of the study | | 3 |
| 1e: Keywords | Include PPI, “patient and public involvement,” or alternative terms as keywords | | PPI, patient and public involvement, co-design, teaching, gastrointestinal, patient education, digital medicine, endoscopy, patient partnership, universal design for learning. |
| Section 2: Background to paper | |  | |
| 2a: Definition | Report the definition of PPI used in the study and how it links to comparable studies | | 4 |
| 2b: Theoretical underpinnings | Report the theoretical rationale and any theoretical influences relating to PPI in the study | | Table 1 |
| 2c: Concepts and theory development | Report any conceptual models or influences used in the study | | 5 |
| Section 3: Aims of paper | |  | |
| 3: Aim | Report the aim of the study | | 5 |
| Section 4: Methods of paper | |  | |
| 4a: Design | Provide a clear description of methods by which patients and the public were involved | | 8-10 |
| 4b: People involved | Provide a description of patients, carers, and the public involved with the PPI activity in the study | | Table 1 |
| 4c: Stages of involvement | Report on how PPI is used at different stages of the study | | 8-10, Table 1 |
| 4d: Level or nature of involvement | Report the level or nature of PPI used at various stages of the study | | 8-10 |
| Section 5: Capture or measurement of PPI impact | |  | |
| 5a: Qualitative evidence of impact | If applicable, report the methods used to qualitatively explore the impact of PPI in the study | | n/a |
| 5b: Quantitative evidence of impact | If applicable, report the methods used to quantitatively measure or assess the impact of PPI | | n/a |
| 5c: Robustness of measure | If applicable, report the rigour of the method used to capture or measure the impact of PPI | | n/a |
| Section 6: Economic assessment | |  | |
| 6: Economic assessment | If applicable, report the method used for an economic assessment of PPI | | n/a |
| Section 7: Study results | |  | |
| 7a: Outcomes of PPI | Report the results of PPI in the study, including both positive and negative outcomes | | 13-16, Table 1 |
| 7b: Impacts of PPI | Report the positive and negative impacts that PPI has had on the research, the individuals involved (including patients and researchers), and wider impacts | | 14-16 |
| 7c: Context of PPI | Report the influence of any contextual factors that enabled or hindered the process or impact of PPI | | 14, Table 1 |
| 7d: Process of PPI | Report the influence of any process factors, that enabled or hindered the impact of PPI | | Table 1, 14 |
| 7ei: Theory development | Report any conceptual or theoretical development in PPI that have emerged  *Note:* by combining PPI principles with universal design for learning principles, we were able to offer more options for inclusion in our resources, allowing the team to learn and collaborate together. This was widely well received by the stakeholder group. | | See note |
| 7eii: Theory development | Report evaluation of theoretical models, if any | | n/a |
| 7f: Measurement | If applicable, report all aspects of instrument development and testing (eg, validity, reliability, feasibility, acceptability, responsiveness, interpretability, appropriateness, precision) | | n/a |
| 7g: Economic assessment | Report any information on the costs or benefit of PPI | | n/a |
| Section 8: Discussion and conclusions | |  | |
| 8a: Outcomes | Comment on how PPI influenced the study overall. Describe positive and negative effects | | 14-17 |
| 8b: Impacts | Comment on the different impacts of PPI identified in this study and how they contribute to new knowledge | | 14-17 |
| 8c: Definition | Comment on the definition of PPI used (reported in the Background section) and whether or not you would suggest any changes | | 8 |
| 8d: Theoretical underpinnings | Comment on any way your study adds to the theoretical development of PPI | | n/a |
| 8e: Context | Comment on how context factors influenced PPI in the study | | 17-18 |
| 8f: Process | Comment on how process factors influenced PPI in the study | | 17-18 |
| 8g: Measurement and capture of PPI impact | If applicable, comment on how well PPI impact was evaluated or measured in the study | | 18 |
| 8h: Economic assessment | If applicable, discuss any aspects of the economic cost or benefit of PPI, particularly any suggestions for future economic modelling. | | n/a |
| 8i: Reflections/critical perspective | Comment critically on the study, reflecting on the things that went well and those that did not, so that others can learn from this study | |  |
